# Why developmental niche construction is not selective niche construction: and why it matters

**DOI:** 10.1098/rsfs.2016.0157

**Published:** 2017-08-18

**Authors:** Karola Stotz

**Affiliations:** Department of Philosophy, Macquarie University, New South Wales 2109, Australia

**Keywords:** developmental niche construction, developmental niche, selective niche, extended inheritance, exogenetic resources, adaptive variation

## Abstract

In the last decade, niche construction has been heralded as *the* neglected process in evolution. But niche construction is just one way in which the organism's interaction with and construction of the environment can have potential evolutionary significance. The constructed environment does not just *select for*, it also *produces new* variation. Nearly 3 decades ago, and in parallel with Odling-Smee's article ‘Niche-constructing phenotypes', West and King introduced the ‘ontogenetic niche’ to give the phenomena of *exo*genetic inheritance a formal name. Since then, a range of fields in the life sciences and medicine has amassed evidence that parents influence their offspring by means other than DNA (parental effects), and proposed mechanisms for how heritable variation can be environmentally induced and developmentally regulated. The concept of ‘developmental niche construction’ (DNC) elucidates how a diverse range of mechanisms contributes to the transgenerational transfer of developmental resources. My most central of claims is that whereas the selective niche of niche construction theory is primarily used to explain the active role of the organism in its selective environment, DNC is meant to indicate the active role of the organism in its developmental environment. The paper highlights the differences between the construction of the selective and the developmental niche, and explores the overall significance of DNC for evolutionary theory.

## 1. Introduction: developmental and selective niche construction

Recent years have seen the emergence of a range of approaches that challenge some basic assumptions of the modern evolutionary synthesis. Several of these have argued that the integration of new causes and processes would amount to an extended evolutionary synthesis [1–5]. One of these approaches is niche construction theory (NCT), which has been heralded as *the* neglected process in evolution (the book's subtitle). NCT argues that organisms actively modify their own environment and thereby influence the selection pressure acting on them and their population. But the construction of an organism's *selective niche* is just one way in which the organisms' interaction with and construction of their environment can have potential evolutionary significance. There is another process of potentially substantial evolutionary influence: the (constructed) environment does not *just select* for new variation, it also *produces* it, in the form of the *developmental niche*. This difference has been highlighted by Piaget 4 decades ago when he theorized about the impact of the behaviour of organism on evolution:

But the central problem remains, for we still have to ascertain how behavior operates here, and whether it intervenes *solely in selection and survival* or is also *a causal factor in the actual formation of morphological characteristics*, as it is suggested notably by Paul A. Weiss’ conclusion that the living organism's organization and hierarchy of the subsystems have a retroactive effect … even upon the functioning of its genome, instead of being simply determined by its functioning. ([6], xi, italics added).

This paper argues for the significance of *developmental* niche construction (DNC) in evolution; clarifying NCT as selective niche construction (SNC) will facilitate distinguishing the two processes from each other. DNC should neither be understood as a subset of NCT, e.g. the developmental production of a selective environment in either evolution or development [7,8], nor as just a cosmetic enhancement of the evolutionary synthesis, without affecting its structure or function. The most central of claims in this paper is that whereas NCT (SNC) explains the active role of the organism in its selective environment, DNC indicates the active role of the organism in its developmental environment. The constructed developmental niche captures the exogenetic (e.g. ecological and social) legacies an organism inherits alongside its genes that together ensure the—potentially modified—reconstruction of the life cycle of the next generation.^1^

The relationship between SNC and DNC may best be compared with the relationship between the modern synthesis and evolutionary developmental biology. The latter provides the developmental mechanisms that connect the phenotype with the genotype, may account for the origin of variation, and highlight the effect of these variations on natural selection without directly affecting the construction of the selective niche. DNC spells out how developmental mechanisms, particularly the construction of a developmental niche, influence the origin of heritable variation and natural selection through the reproduction of a developmental system at the individual level. In other words, DNC is concerned with the origin of potentially adaptive, heritable, phenotypic variation. So while standard evolutionary theory assumes that all adaptations are the result of natural selection at the population level, this paper addresses the possibility that development can account for the creation of adaptations without invoking selection—a point that has been dubbed the arrival or origin of the fittest, rather than its survival [9].

Proponents of an extended evolutionary synthesis are used to being rebuffed by comments of defenders of the status quo that the founders of the modern synthesis have been well aware of all of the phenomena and processes that are now being cited as support for the need of an extension. Where that was the case, however, that was often only to marginalize their importance, as was the case of Simpson's belittlement of Waddington's genetic assimilation as ‘Baldwin Effect’, named after a disgraced psychologist from the turn of the century [10,11].^2^ Nobody can dispute, however, that development, particularly the developmental system comprising the organism within its developmental environment, was the most neglected process in evolution. This neglect was defended on the ground that development was entirely under the control of the genetic programme, an outcome of random mutations and natural selection. To the point that the phenotype was influenced by development and environment, it was deemed evolutionarily insignificant because only genes were regarded as having heritable effects on the fitness of an organism. The defenders of the modern synthesis would be correct to point out that it is not new that organisms shape their environment, or that the parent's phenotype influences the phenotype of their offspring, but this has rarely been stressed with any real urgency, nor has it led to a change of the standard way evolutionary theory is conceptualized in textbooks.

The theory of DNC integrates development as a contingent, constructive and emergent process of the interaction between developmental resources and the ecological context with the idea of inheritance as the transfer of essential developmental resources vital to the reconstruction of the next generation's life cycle. Such a theory needs to have a balanced account of the robustness of the organismal organization, the generation of novel variation, and its inheritance to the next generation. Arguably, its most critical component is the concept of extended inheritance that goes beyond the transmission of DNA sequences to accept the evolutionary significance of environmentally induced and developmentally regulated origin of novel variation.

The following section starts with a juxtaposition of two different niches, namely the selective and developmental niche, and highlights their differences (§2). Section 3 introduces the theory of DNC, traces its origin, points out its central idea of extended inheritance and how it extends to human niche construction. In §4, I will discuss the main distinguishing features between the two accounts of niche construction, including the divergence between my account of DNC and NCT's take on what they termed DNC as well. Section 5 shows DNC's evolutionary significance by situating it among a list of proximate causes in evolution and how DNC can be employed to answer some of the pressing questions that evolutionary theory attempts to answer. The paper closes with a conclusion and a future outlook.

## 2. Development and two kinds of niche construction

Originally, the term ‘niche’ was synonymous with the *ecological niche*, which refers to the ecological role of an organism in, and its relationship to, its ecosystem. Recent discussions within evolutionary theory have invoked two new and distinct concepts of the niche, which are the main concern of this paper.

### 2.1. Parameters of a niche

A niche is a relativistic and dynamic concept that needs to be defined relative to its causal relationship with its inhabitants. The selective niche is defined by the environmental parameters which have a causal influence on the differential survival and reproduction rate of organisms. It figures in NCT as that part that is created by the action of the developing organism. In other words, the selective niche, produced by the capacity of the developing organisms to modify sources of selection in their external environment, in combination with the part that is beyond the control of the organism, is coextensive with the set of selective pressures on the population.

The developmental niche by contrast is defined by the environmental parameters that play a role in the modification and reproduction of the life cycle. These parameters are the environmental cues inducing the development of plastic phenotypes. That part of the developmental niche DNC is concerned with is created by the action of and interaction between parent, offspring and the social group and trans-generationally transmitted to offspring to causally influence the development of the offspring's phenotypic traits. The developmental niche is a multi-dimensional space of environmentally induced and developmentally regulated, heritable resources that scaffold development. It provides a link between the generations through mechanisms that promote the transitions for young and adult species-typical development [12,13].

The developmental and selective niche of a population can but do not have to overlap. Think, for instance, about a predator-induced polyphenism where the exposure to a predator induces the development of a defence in the prey. In the waterflea, actual experimental studies have revealed that the presence of the predatory larvae of the fly *Chaoborus* causes the development of a spiked helmeted morph that is more successful in surviving the predator [14]. While at first glance the two niches may appear to overlap, the cue of the developmental niche to induce the predator-protected morph is not the predator itself but chemicals released by the predator. It is, however, not these so-called kairomones, but the predator itself that is the defining parameter of the selective niche. Offspring of *Daphnia* parents exposed to the predator may even develop the defensive morph in the absence of predators, a maternal effect caused by some unknown epigenetic mechanism defining the offspring's developmental niche (see [15, p. 27], for a summary and citations of original research). This latter mechanism is an example of a ‘predictive adaptive response’ (PAR), although in the above case characterized by the absence of the predator, it would amount to a ‘mismatch’ between the predicted response and the experienced selective niche (see [9]).^3^

According to Alex Badyaev, natural selection results from ‘a mismatch between the environment of development and the environment of functioning’ [16, p. 1924], which influences the outcome of development and determines how the organism fares in terms of survival and reproductive success, respectively. In other words, the more the developmental and selective niche overlap, and hence the more the developmental niche is causing an adaptive phenotype, the less the selective niche causes negative selective pressure on the organism or population. But here, and likely many other cases, the environmental cue for the development of a certain adaptive phenotype may not be a defining parameter of the selective niche, even though they are correlated. The niche of a nest, for example, may be equally important for growing up and for survival, although again, it would still need to be established if the parameters of the nest most protective against harsh environmental conditions or predation are the same as those providing the right temperature exposure for development or the best affordances for learning.

The developmental niche has been likened to a link between parent and offspring. In rats, the developmental niche for the pups called ‘dam’ is at the same time the developmental niche of the dam marking her transition to a nursing mother. The parameters important to the pups are the grooming and provisioning provided by the mother that is the necessary stimulation for neurological development. The licking of the pup's urogenital area at the same time releases the pups' urine which partially compensates the mother for her loss of fluid and electrolytes during nursing. The developmental niche of the mother overlaps here with her selective niche as the investment beneficial to offspring can be costly to parents. Parent–offspring conflict is a tug-o-war between the differing demands of parent and offspring [17,18], or the developmental and selective niche of offspring and parent.

### 2.2. The selective niche: selective feedback through ecological inheritance

NCT is one of two theories with reference to the niche designed to put the active organism back at the centre of evolutionary theory. It refers to the process by which an organism alters its own selective environment and hence influences its own and its species’ selection pressure. It suggests that rather than populations of organisms passively adapting to a changing environment, they actively construct their environment—their *selective niche*—and thereby change the dynamics of evolution. Niche construction shapes the selection pressure of the population, and can result in the ecological inheritance of its selective niche; both of these processes affect the fitness of future generations [19,20]. It goes back to Lewontin's insistence that organisms are not just the passive outcomes of natural selection but subjects of their own evolution [21].

The *selective* niche is defined by the parameters that determine the relative fitness of competing types in a population. In SNC, generations partly construct the selection pressures that act on future generations. As an example, some recent work on human evolution has emphasized the role of ecological niche construction in human evolution: the evolution of the unique characteristics of human psychology and social structure has been substantially driven by the selection pressures created by earlier psychological and social structures [22–24]. The ecological niche of humans overlaps substantially with their cultural niche that features in models of gene–culture coevolution, with genetic and cultural inheritance involved in a complex feedback loop via natural selection.

### 2.3. The developmental niche: a formal account of exogenetic inheritance

Another aspect of niche construction is that development is dependent on a rich *developmental niche*, constructed in interaction with parents, other conspecifics, the physical and biological environment, and cognitive artefacts from tools to languages. Just like the construction of the selective niche, the developmental niche plays an important role in evolution: the environment not just *selects for*, it also *constructs* new heritable variation (see [13, p. 369]). The developmental niche is defined by the parameters needed to ensure the (re)construction and modification of the evolved life cycle, which often do not coincide with the parameters as the selective niche.

The developmental niche figures implicitly in developmental system theory (DST), a theory developed in the 90s that focuses on the active role of the organism in evolution [25,26]. At the centre of DST lies the life cycle of a developmental system, which comprises the organism and its relationship to its developmental environment, the developmental niche. The concept of the developmental niche is designed to integrate and formalize the non-genetic (exogenetic) yet heritable factors influencing an organism's development. It is therefore the *evolved* developmental niche that provides channels of sustenance for the developing organism, such as nutrients, warmth, insulation, and behavioural and social stimuli. It ‘nurtures’ the offspring in the form of resources, stimulation and affordances for development, i.e. it gates what is available to be learned. The evolved developmental niche defines several pathways by which effects of experience on the parental generation can be transmitted to later generations [27–32].

The concept goes back to the ‘ontogenetic niche’ coined by developmental psychobiologists West & King [12,13,33]. In the latest formulation of the concept, the developmental system consists of epigenetic resources inherited through the germline, and an exogenetic developmental niche, which contains *reliably* but *flexibly* inherited physical, social, ecological and epistemic resources needed to reconstruct, or modify, that developmental system [34]. These resources can be actively constructed by the parents (producing the ‘parental effects’ of quantitative genetics) or by the larger group in interaction with the offspring, or sourced passively from the environment. Wherever they come from, if there exists an evolutionary explanation for the interaction of the evolved developmental system with an internal or external resource then that resource is part of the system. What evolves by natural selection is a relationship between a system and each of its resources.

The developmental niche has two fundamental functions as commonly understood in evolutionary biology.^4^ One function is to facilitate the robust and reliable development of species-typical traits, the other is their plasticity that leads to substantial diversity among a species (see §5). So what explains the *typicality* of a species is the developmental systems dynamics within what one may call ‘normal’ parameters, some of which are provided by pre-existing physical and developmental constraints. The rest are ensured by reliably inherited resources, which are not just the genome but essential environmental resources that assist, among other functions, in the species-typical expression of the genetic factors. Reciprocally, this expression in turn assists in the creation of some of the essential environmental resources. These stable resources are also what partially explain the *fixity* of a species.^5^

But an account of any species also needs to embrace and explain diversity: here the second function of the developmental niche comes in. Beyond ensuring reliable, species-typical development, the developmental niche also provides input to developmental plasticity (e.g. [35]). Plasticity is often defined in terms of a genotype's ability to produce different phenotypes in response to the environment. It would be more accurate, however, to say that the shape of the norm of reaction is a property of the whole developmental system. So what explains diversity are differing developmental systems dynamics supported by modifications in the developmental niche. In other words, diversity results primarily from the interaction between the evolved developmental system and a wide range of environments, including novel environments. DNC therefore provides dependability, but also adaptive flexibility, in the provision of necessary developmental resources.

So to summarize: the developmental and the selective niche differ epistemically: DNC and NCT answer different questions. DNC is about the production of (adaptive) variation, while NCT is about the selection of variation. The developmental and the selective niche also differ ontologically: in cases where the developmental niche concerns the production of adaptive traits, it would influence the outcome of selection through the production of an adaptive phenotypic response, rather than through modifying the selective niche.

## 3. Developmental niche construction

The construction of the developmental niche relies heavily on the extragenetic inheritance of developmental resources. This heterogeneous process includes maternal and paternal parental effects, which cannot be reduced to the influence of parental genes or gene product on their offspring, but include all processes of care for the offspring. These comprise differential provisioning of resources, preference induction (oviposition, imprinting on food, habitat and mates) and social learning, to name just a few [36,37]. Both forms of niche construction are a form of ‘extended inheritance’—albeit the role of ecological inheritance for NCT and DNC is different (§4 and table 1). Inheritance systems have evolved to make the transmission of crucial information from parents to offspring more reliable. A reliably reproduced developmental system is the result of the reliable provision of a wide range of developmental resources necessary to reconstruct the organism's life cycle. But transmitted resources are sensitive to the parents' environment and can be modified accordingly to prepare the offspring to changed ecological circumstances. Beyond DNA, additional and equally necessary resources are epigenetic modifications, cellular structures, nutrients, gut organisms, parental care and for many species the social environment. Organisms have developed a range of strategies to construct and modify the developmental niche for their offspring to guide the developmental process.

**Table 1.** Areas of difference between NCT and DNC.

areas of difference	NCT	DNC
(*a*) role of development in evolution	source of natural selection	source of phenotypic variation
(*b*) domains	mainly external environment	DNC both internal and external
(*c*) inherited resources	mainly a dual inheritance model of genetic and ecological/cultural resources	interacting and codependent channels of inheritance
(*d*) role of inheritance	inherited selection pressure act on offspring population via natural selection or learning environment	inherited developmental resources construct offspring phenotype, which includes learned behaviour
(*e*) reciprocal reasoning	organisms alter environment to change their own selection pressure	organisms alter environment to change input into their own or their offspring's development

West & King said ‘Ask not what's inside the genes you inherited, but what your genes are inside of’ [12, p. 552], particularly extra-organismal. Looking at the enormous complexity of gene expression of eukaryotes that reveals a very flexible and reactive genome open to many intra- and extra-organismal environmental influences, it was simply a matter of time before some systems found ways to manage aspects of their own developmental environment. It is not so much the particular gene you inherit that counts but when genes are switched on and off, which parts of the DNA sequence will be transcribed and spliced and in which combination, which will be edited at certain nucleotides, which will be translated and at what rate and what will be the post-translational modifications (see [30] for a more detailed description).

DNC is the control of the next generation's developmental environment through extended inheritance. Parental activity can facilitate, guide and entrench social learning, which in the case of humans and higher animals falls under the rubric of the cultural transmission of information. What all of these above cases of inheritance through environmental construction have in common is making the transmission of crucial information more reliable through the reconstruction of the developmental niche, but through the phenotype's latent plasticity also more ecologically responsive in a modified developmental niche.

There have been repeated attempts to reduce all of these mechanisms to the action of inherited or parent-of-origin genes, so that ultimately the real causes are all genetic. This special pleading fails in the light of the discovery that development relies less on the existence of genes in an organism than on the regulated expression of these genes, which ultimately depends on a host of environmental factors. Wherever there are genes, there are extragenetic factors necessary for their regulated expression.

### 3.1. A new synthesis of epigenesis, evolution and extended heredity

What a new account of development, and of DNC, really has to accomplish is to provide a framework that integrates a complex set of heterogeneous factors into a system of developmental resources all of which are more or less reliably but yet flexibly reproduced in succeeding generations but none of which belong exclusively to either ‘gene’, ‘organism’ or ‘environment’ [38, p. 557]. Its contextualization of genes should obviate ‘even naïve temptations toward gene/environment dichotomies and … will open up a very rich area of empirical investigations to examination and conceptualization in developmental-system terms' [39, p. 85]. The important systems features of such a view are the rejection of dichotomous descriptions of behaviour in favour of a full analysis in terms of continuing interaction between, and the joint determination by, heterogeneous developmental resources. A central feature of such a view is extending inheritance to include other factors than DNA—factors formerly thought of as ‘environmental’ or ‘experiential’—if they are reproduced or ‘passed on’ to succeeding generations [40, p. 6].

DST describes evolution as construction where organisms are not independent of or just passively dependent on their environments; they and their parental generation actively construct their developmental and selective niches which are an integral part of the whole developmental system. Obviously, the more extended development is, and the more behaviourally plastic an organism, the more rampant and important are its DNC activities. This is most obvious in humans.

### 3.2. An example: the developmental niche of humans

How does the developmental niche influence human development? Human babies are needy. They are born prematurely in comparison to other primates, meaning that for several months postnatally, relative to other primates, human babies share characteristics of fetuses rather than of infants in those other primates [41]. Comparing brain size at birth among primates, humans should be born at 18 months of age, despite gestation period tracking female body mass fairly closely. A large part of brain development takes place outside the uterus, influencing human offspring epi- and exo-genetically much more postnatally than their ape cousins, which makes the early niche fundamental for human development. Over the course of human evolution, as brains became bigger and human infants more immature at birth, human childrearing practices evolved in tandem with these changes to ensure the survival of the helpless infant. As bipedalism, haemochorial placenta, large brains and the need for a great amount of learning after birth emerged, human evolution intensified parental care: ‘Only with intensified parental care in response to greater helplessness of the infant could selection favour the evolution of a large brain in a bipedal animal’ [41, p. 33]. The evolution of a more complex and resource-demanding developmental niche has been a key feature of human evolution.

While a developed treatment of human nature is beyond the scope of this paper, I claim that human nature resides partly in the human developmental environment [42]. We are a species that is particularly strongly influenced by niche construction, both SNC over evolutionary timescales and DNC over ontogenetic timescales. A concept of nature according to which what is natural must come from the inside is particularly unsuitable for such a species. Imagine trying to determine the real nature of an ant, another powerful niche constructor, by removing the influence of the nest on the developing egg and embryo. The result would be either dead or biologically meaningless, and so it is for humans. The concern is that when the developmental niche is not provided, the offspring will not develop in a species-typical manner.

As social mammals, humans have an intensive developmental niche for their young— soothing perinatal experience; warm responsive care; nearly constant physical touch (carrying, cosleeping); years of breastfeeding; free play in the natural world [43]. The human developmental niche became more intensive because of the immaturity of the neonate, adding to the social mammalian practices a positive climate of mother–infant dyad support and multiple adult carers [44]. All these practices have known epigenetic and plasticity effects on neurobiological systems and long-term well-being of the child (for reviews, see: [45,46]). The developmental niche has powerful effects on the type of human nature one develops, as notable among societies who routinely provide it—small-band hunter–gatherers (e.g. [47]), the type of society in which the human genus spent 99% of its genus history [48]. Recent empirical studies also show the developmental niche's relation to adult mental health, sociality and morality [49,50].

The developmental niche presents a twofold link, first between the young and adult form by scaffolding its development, and second between generations: ‘Members of both generations must act to realize their investment as parents or inheritances as offspring. The niche is thus a way of life and is the study of behavioral ecology’ [13, pp. 46–47]. All inheritance equates with the dependable, transgenerational transmission of crucial information, but extended inheritance leads to ‘transgenerationally extended plasticity’ in the form of developmentally induced heritable epigenetic variations. Thus coming to terms with an animal's nature means transcending features of similarity, universality and fixity in order to integrate diversity, plasticity and adaptability.

## 4. Differences and conflations

The main difference between NCT and DNC is their contribution to two different creative forces in evolution: the origin of natural selection versus the origin of heritable phenotypic variation (see §5). To entangle the differences of perspective between NCT and my account of DNC, one can distinguish several other areas of difference that summarize the claims made in this paper so far (table 1).

(i) The effect of development on evolution: the main contrast between the two kinds of niche construction is their understanding of the main causal influence the process of development has on evolution: while DNC is interested in the source of phenotypic variation, NCT looks for the source of natural selection (see §§1 and 2). (ii) The domains concerning the environment: while NCT's main concern is with the external (ecological and cultural) environment, DNC addresses both the internal (epigenetic, cognitive) and external (ecological, socio-cultural, epistemic and symbolic) environment of the organism. (iii) The systems of inheritance: NCT subscribes mainly to a dual inheritance model of genetic and ecological/cultural inheritance, with genetic inheritance being still seen as primary, albeit modified by the supplemental system of ecological and cultural inheritance. One should add that recently proponents of NCT have begun to recognize the importance of epigenetic inheritance as well (although without clarifying its role for the process of niche construction, e.g. 8). DNC by contrast is a framework for integrating a diverse range of exogenetic inheritance systems (see point 2). (iv) The roles played by extended inheritance: while for the former, inherited selection pressure acts on the offspring population via natural selection or learning environment, for the latter, inherited developmental resources construct the offspring phenotype, which includes learned behaviour. (v) Reciprocal reasoning: according to NCT, organisms alter the environment to change their own selection pressure, according to DNC, organisms alter the developmental environment to change the input into their own or their offspring's development.

Against this quite substantial divergence between the two accounts, they are united by their view of the evolutionary significance of a fluid organism–environment boundary, and their focus on the importance of an active developmental system for the evolutionary process. Both NCT and DST/DNC understand the developmental system as the whole life cycle of the organism–environment system, which is not just the passive object of evolution but instead the subject or co-director of its development and evolution [51]. ‘All developmental processes that modify the organism–environment relationship are recognized as evolutionarily causal’ [7, p. 555].

A complication of just pointing to the differences between the two accounts arises through the fact that ultimately the level of phenotypic pre-adaptiveness to the environment of functioning influences the selection pressure experienced by the organism, so that even DNC is indirectly part of the process of SNC. Therein may lie the reason for the many conflations of the two processes within the literature, although this is hard to determine because the different causal pathways towards the creation of natural selection are rarely spelled out. One way for phenotypic variation to adaption is adapting the organism to its environment, thereby reducing natural selection. Another route for the production of phenotypic variation to influence the selection pressure acting on an organism is directly through the production of niche constructing behaviour, potentially adapting the environment to the organism. This second route is still the main focus of Flynn, Laland, Kendal and Kendal's target article ‘Developmental niche construction’, albeit now with selection acting on learning:

Learning and development can be of considerable importance to evolution because learned knowledge can guide niche construction (…) Niche construction modifies selection not only at the genetic level, but also at the ontogenetic and cultural levels as well, to facilitate learning and mediate cultural traditions …. [8, p. 299]

It is this reason why what Flynn and co-workers label DNC and the process called DNC proposed here—in line with [12,28,31]—are distinct processes. It may also be the reason why proponents of NCT often seem to conflate, or least seem unclear about, the underlying difference between NCT and DNC, as seen below from a paper comparing NCT and evo-devo:

The incorporation of ecological inheritance into evolutionary biology has consequences for development. It means that in each generation, offspring inherit a local *selective environment* that has, to an extent, previously been modified, or chosen, by its niche-constructing ancestors. (…) In standard evolutionary theory, the development of organisms begins with the inheritance of a ‘start-up kit’ of genes: in niche-construction theory, it begins with the inheritance of a ‘start-up niche’. ([7], p. 556)

The start-up niche would be the developmental niche scaffolding the development of the system before any selection can act on it! While referring to a selective environment, their examples that follow this quote are of parental effects on offspring phenotype through oviposition and the allocation of nutritional resources and protective chemicals, which are a clear example of DNC as proposed here. Another example:

Thus, development closely resembles evolution (…) in that they are both ‘interactionist’ processes (…), reliant on reciprocal causation. Both involve organisms responding to their *selective environments,* and modifying their *selective environments* by their niche-constructing activities. [7, p. 557]

Again, the authors refer to the selective environment, but then follow up with examples that clearly show how a created niche may ‘modify and permit the development of the organism’.

It then becomes clear that when they talk about how ‘developmental niche regulation’ or the ‘developmental process of niche construction’ can influence evolution that they do not talk about the developmental niche, but the construction of the selective niche during development. While their example of a symbioses between gut organisms and their host serves to show the microbe's SNC, it could also be understood as a developmental niche influencing the developmental gene expression of the host [6, pp. 557–558].^6^

## 5. Implications of developmental niche construction for an extended synthesis

There are now a myriad of theories that stress the importance of development for understanding evolution. A variety of accounts fall under the umbrella of developmental accounts of evolution: developmental systems theory (DST; [25,52]) and DNC, which includes and takes serious the idea of extended inheritance [36], evolutionary developmental biology (evo-devo; e.g. [53]), developmental evolution [54], facilitated variation [55,56], ecological developmental biology (eco-devo; [15,57,58]), phenotypic [35] and developmental plasticity [59], and niche construction [20]. One way to distinguish between them is by asking which mechanisms are treated as the main ‘creative’ force of evolution. According to Gould [60], Darwin was the first to acknowledge natural selection not just as a negative and conservative force, but a positive and creative force in promoting evolutionary change. While NCT and gene–culture coevolution focuses on the origin of natural selection, most approaches focus on the origin of adaptive variation, evolutionary novelty and innovation as an alternative positive force.

### 5.1. Proximate causes and processes in evolution: two creative forces

While traditional evolutionary theory has often treated natural selection as the main or even only creative force in evolution, Darwin had originally acknowledged the interplay of two forces that shape evolution: the origin of adaptive and heritable variation which is providing the raw ingredient on which natural selection can act, and natural selection. While Ernst Mayr has famously claimed that only ultimate, not proximate causes in biology address ‘why’ questions of evolutionary biology, in recent times, this claim has been seriously questioned by arguing that proximate causes derived from ontogenetic processes are of relevance in answering questions of interest in evolutionary theory. In other words, both so-called creative forces are affected by a range of proximate causes (table 2). The origin of adaptive variation is addressed by diverse accounts studying a range of phenomena: (i) phenotypic accommodation and developmental plasticity,^7^ (ii) developmental bias on the origin of variation and novelty, (iii) facilitated variation, internal selection and self-organization, (iv) genetic assimilation (canalization) and accommodation (plasticity) (which Sir Patrick Bateson calls ‘adaptability driver’) and last but not least (v) DNC and exogenetic inheritance. The origin of natural selection is thought to be influenced by phenomena like: (i) the existence of populations of complex adaptive systems, (ii) developmental bias on the action of natural selection, and last by (iii) niche construction, ecological inheritance and cultural evolution.

**Table 2.** Proximate causes behind the two creative forces of evolution.

origin of adaptive variation	origin of selection
— phenotypic accommodation/developmental plasticity — developmental bias on variation/novelty — facilitated variation, internal selection, self-organization — adaptability driver, genetic assimilation and accommodation — developmental niche construction and exogenetic inheritance — cultural evolution	— complex adaptive systems with differential abilities to reproduce — developmental bias on natural selection — the shape of particular traits and how they interact with particular processes in the environment — selective niche construction and ecological inheritance — cultural evolution

Kirschner & Gerhard, the originators of the theory of facilitated variation, point out that the two creative forces, and by fiat the processes of niche construction (see also §3.3) are actually not independent of each other but are independent causes of a single outcome are losing importance to the extent that the other is gaining it.

The cardinal issue in evolution is the origin of complex and heritable variation. Although selection has preoccupied evolutionary biologists, the study of the origin of variation and novelty has idled (…) The more important … the environment in determining the kind of variation, the less was its importance as a selective and creative agent. (…) Thus, the efficacy of selection would depend on the nature of phenotypic variation. [55, p. 8, 3, 13].

As mentioned in the Introduction, one of the main reasons why proximate causes gain in their power to address evolutionary questions is that the hold of the metaphor of the ‘genetic program’ is weaning. It is now clear that epigenetics, which relays environmental information to the genome, not only regulates when and where the specificities encoded in the library are to be expressed. It also substantially augments the information of the literal coding sequence. A strange aspect of the management of genetic information is that the epigenetic control system—which Paul Davies likens to ‘an emergent self-organizing phenomenon’ [61, p. 42]—does provide more than just a supervising function on the expression of the specificities encoded in the DNA. As the information encoded in the DNA does not entail a complete set of instructions for which biomolecules shall be synthesized, the epigenetic control system amplifies the information of the literal code [62–64]. Therefore, we can say that a substantial amount of information needed to construct an organism is derived from elsewhere, such as the organism's environment (see [65]). This information augments or amplifies the information inherited via the genome [30,66].

### 5.2. Evolutionary questions

This section is concerned with what kind of questions evolutionary biology is concerned with and which kind of answers would provide their explanations. The most common questions acknowledged are (i) the origin of species and species diversity and (ii) the origin of fit between organism and the environment. According to Pigliucci & Kaplan, evolutionary biology should explain ‘the origin, spread, and maintenance of phenotypic traits, as well as the developmental pathways that reliably (re)produce them’ [67, p. 112]. Another question is the modification of traits. One major importance of DNC lies in its integrative power: it combines ideas of the active organism altering its environment (niche construction), a systems view of development (DST), extended (exo-genetic) inheritance and the origin of novelty (‘evo-eco-devo’ and phenotypic plasticity). Because of the role it plays in explanations of all of the above-mentioned evolutionary questions, its integration into mainstream evolutionary theory will be an important part of the continued refinement of this continuously evolving field.

The process of DNC has evolutionary significance because it has impact on phenotypes through its new construction, reconstruction, maintenance and modification. Hence it contributes to answer:

(i) the origin of a trait by introducing new epi- and exogenetic resources for novelty and innovation and proposing mechanisms of how developmental, self-organizing processes contribute to the emergence of novel phenotypes. These mechanisms together with (iv) can also be invoked to answer the question of the origin of species and species diversity; (ii) the spread of a trait by showing how organisms or their parental generation co-construct a developmental environment which contributes in the production of adaptive variations; (iii) the maintenance of a trait through processes that create and recreate a species-specific environment; (iv) the modification of a trait by making the transgenerational transmission of exogenetic resources as ecologically open as possible; and (v) the developmental pathways that reliably (re)produce traits through transgenerational stability that extend the inheritance beyond the transmission of genetic material with the reliable availability of necessary developmental resources through multiple mechanisms of reproduction or transmission of developmental resources. This last question invokes the developmental control of heredity (table 3).

**Table 3.** Question asked by evolutionary theory and answers provided by DNC and extended inheritance.

questions in evolutionary theory	answers in evolutionary theory
(*a*) origin of a trait	new epigenetic and exogenetic resources for novelty and innovation developmental, self-organizing processes contribute to the emergence of novel phenotypes
(*b*) spread of a trait	organisms or their parental generation co-construct a developmental environment which contribute in the production of adaptive variations
(*c*) maintenance of a trait	processes that create and recreate species-specific environment
(*d*) modification of a trait	ecologically open transgenerational transmission of exogenetic resources
(*e*) reliable reproduction of trait	transgenerational stability through the reliable availability of necessary developmental resources through multiple mechanisms of reproduction or transmission of developmental resources developmental control of heredity
origin of species and species diversity	see mechanisms supporting (*a*) and (*d*)

## 6. Conclusion and future outlook

This paper stresses the distinction between two quite different ways in which organisms modify their living environment: developmental and SNC. The well-known NCT is mostly about how organisms modify their exposure to natural selection, while in addition it has recently paid attention to how humans influence selection to facilitate learning. The authors [8] termed this latter process DNC, which is unfortunate because it conflates this process with a process I have called DNC [5,28,30,68] that is more in line with the spirit of the term ontogenetic or developmental niche used in developmental psychobiology and psychology as well as parental effect research [12,31,33,69,70].

Flynn and co-workers' work on DNC is an exercise in building bridges between NCT and developmental psychology, reminiscent of the work of Stotz’ ‘Human nature and cognitive–developmental niche construction’ [28] in connecting DNC to the tradition of situated, embodied, embedded, enactive and extended cognition. This points to the potential benefit of this theoretical and conceptual work. As Wagner has pointed out

*Any concept is only as good as the research program it inspires*. Thus, whether an idea is ‘good’ depends on the skill of its proponents to turn it into a productive research program; concepts should play the role of inspiring and guiding progressive empirical and theoretical investigations. ([71, p. 340], italics in original).

Therefore, it remained to be seen how fruitful such a cross-fertilization turns out to be. One way in which theoretical work on DNC and exogenetic inheritance may be useful to psychological research is by the conceptual tools for quantifying the impact of exogenetic resources on development ([72], see also [65]). Also, DNC can function as a useful framework to integrate the results from different research areas concerned with epigenetic and exogenetic inheritance and parental effects. Last, such an integration may add weight to the impact of these research areas on the construction of a broader evolutionary synthesis.

## Endnotes

1 This paper will be mostly concerned only with those parts of the respected niches which are actively constructed by organisms.

2 What Waddington saw as an evolutionary significant process that could fast-track adaptations, Simpson dismissed as a mere coincidental effect of little importance.

3 The scope of the paper forbids the introduction of more empirical examples despite their importance to underline the theoretical points of the paper.

4 A trait's function causally explains its existence in the population via the mechanism of natural selection.

5 In addition, there are homeostatic and self-organizing mechanisms of the organism that buffer against internal (genetic, epigenetic, metabolic) and external perturbations. These are invoked when we talk about canalization.

6 There exist many more examples of these types of conflation mostly in [7,8] that cannot be spelled out and discussed in detail in this short paper.

7 See Sonia Sultan's contribution to this issue on the question of how much this competence is due to past selection. In each case, this would be an empirical question.
